# Barriers and facilitators to implementing the birth preparedness and complication readiness strategy: midwives’ perceptions

**DOI:** 10.1186/s12884-025-08307-3

**Published:** 2025-11-10

**Authors:** Deborah Tolulope Esan, Kenneth Akintunde Onilude, Carlos Guillermo Ramos

**Affiliations:** 1 Faculty of Nursing Sciences, College of Health Sciences, Bowen University, Iwo, Nigeria; 2 Department of Pediatrics, University of California, San Diego, USA

**Keywords:** Birth preparedness, Complication readiness, Midwives, Barriers, Facilitators, Maternal health, Antenatal care

## Abstract

**Background:**

Maternal mortality remains a major public health concern in Nigeria, largely due to delays in accessing skilled care. Birth Preparedness and Complication Readiness (BPCR) is a strategy designed to improve timely access to care. This study explores midwives’ perspectives on barriers and facilitators to BPCR implementation in Ogbomoso, Nigeria.

**Methods:**

A qualitative study was conducted involving in-depth semi-structured interviews with 14 purposively selected midwives from four healthcare facilities in Ogbomoso. Data were analyzed using Tesch’s eight-step thematic analysis approach.

**Results:**

Barriers to BPCR implementation included poverty, cultural resistance, health illiteracy, staffing shortages, geographical barriers, and limited male involvement. Facilitators included visual health education, support from community and religious leaders, family involvement, peer support among midwives, and NGO assistance.

**Conclusion:**

Midwives face complex barriers in promoting BPCR in resource-limited settings. Strengthening health systems, enhancing community engagement, and improving training and support for midwives are key to improving maternal health outcomes.

## Introduction

Maternal and neonatal mortality continue to be critical public health issues, particularly in low- and middle-income countries (LMICs) such as Nigeria. Globally, an estimated 295,000 women died during and following pregnancy and childbirth in 2017, with the vast majority of these deaths occurring in sub-Saharan Africa and Southern Asia [25]. Nigeria alone accounts for approximately 20% of global maternal deaths, with a maternal mortality ratio (MMR) estimated at 512 deaths per 100,000 live births, which is among the highest worldwide [23, 25]. This high burden of maternal mortality is largely due to preventable causes such as hemorrhage, hypertensive disorders, infections, and obstructed labor [7]. Neonatal mortality remains a major contributor to under-five mortality, with an estimated neonatal mortality rate of 33 deaths per 1,000 live births in Nigeria [22].

In response to these challenges, the strategy of Birth Preparedness and Complication Readiness (BPCR) has been recognized as a vital approach to reduce delays in accessing maternal health services and improving maternal and neonatal outcomes [8]. BPCR is a comprehensive framework that encourages pregnant women, their families, and communities to plan for normal childbirth while anticipating potential obstetric complications. It promotes the identification of a skilled birth attendant, selection of a birth facility, arrangement of transportation, saving of funds for emergencies, and awareness of danger signs during pregnancy, childbirth, and postpartum periods [7, 24].

The “three delays” model—delays in decision-making to seek care, reaching health facilities, and receiving appropriate care—has been foundational in shaping BPCR interventions [20]. BPCR aims to address these delays by increasing awareness, improving access, and facilitating timely use of skilled obstetric services [8]. Evidence from various LMICs suggests that women who practice BPCR are more likely to seek skilled care and experience better maternal and neonatal outcomes [5, 21]. In Ethiopia, for example, women who were well-prepared for birth were significantly more likely to deliver at health facilities and had reduced risks of obstetric complications [21].

In Nigeria, studies have demonstrated varying levels of awareness and practice of BPCR. Iliyasu et al. [7], in their study among pregnant women in northern Nigeria, reported that although awareness was moderate, actual preparedness in terms of arranging transport or identifying a skilled birth attendant was low. Similarly, Ajibade, Afolabi, and Adeniran [4] found that socio-economic status, education level, and urban residency were significant predictors of better BPCR practices, with rural women less likely to be adequately prepared. This disparity highlights persistent inequities in maternal health services access and utilization between rural and urban populations [4].

Socio-cultural factors play a substantial role in influencing BPCR adoption. Traditional beliefs that childbirth is a natural event not requiring medical intervention persist in many Nigerian communities, limiting women's utilization of skilled care and BPCR measures [18]. Male partners often dominate decision-making regarding healthcare seeking, and financial dependency on husbands can restrict women’s autonomy to plan and save for delivery [14]. Preference for traditional birth attendants (TBAs) is prevalent due to their cultural acceptability, affordability, and accessibility, especially where formal health services are scarce [2].

Health system barriers further complicate the implementation of BPCR in Nigeria. The health sector suffers from inadequate funding, insufficient infrastructure, shortages of skilled health workers, and poorly functioning referral and emergency transport systems [16]. Primary healthcare centers (PHCs), which should serve as the first line of maternal care, often lack essential equipment, trained personnel, and reliable power supply [17]. Consequently, pregnant women in rural areas face difficulties reaching timely and appropriate care, which BPCR aims to mitigate [23]. Importantly, studies highlight that midwives themselves experience workplace adversities such as staff shortages, limited resources, and unsupportive work environments, which directly constrain their ability to promote BPCR effectively [1, 19]**.** Soubeiga et al. report that organizational factors, including staffing ratios, are critical barriers to implementing Birth Preparedness and Complication Readiness (BPCR) programs, which adversely affect maternal and neonatal care outcomes [19]. Additionally, research by Lukasse and Henriksen emphasizes that midwives’ express concerns regarding inadequate resources and poor staff mix, associating these conditions with fears of adverse events during patient care [10]. Such an environment directly impacts midwives' professional well-being and their capacity to advocate effectively for BPCR initiatives.

Midwives are central to the promotion and implementation of BPCR. As frontline providers of antenatal, delivery, and postnatal services, midwives provide education on BPCR components and encourage positive health-seeking behaviors [3]. However, midwives face challenges such as staff shortages, heavy workloads, lack of continuous training, and limited resources that reduce their ability to effectively deliver BPCR education [11, 26]. Studies have shown that when midwives receive adequate training and support, BPCR education leads to improved preparedness among pregnant women [13]. In South-east Nigeria, midwives emphasized the importance of person-centered maternity care but reported systemic constraints such as overcrowding, weak referral systems, and lack of supportive supervision that undermine effective BPCR counselling [15].

Globally, the WHO and other stakeholders emphasize the integration of BPCR into routine antenatal care services to reduce maternal and neonatal mortality [24]. Evidence from systematic reviews indicates that comprehensive BPCR interventions contribute to a significant reduction in maternal mortality by encouraging early detection of danger signs and prompt care-seeking [12]. Evidence from Nepal also demonstrates that strengthening BPCR through skilled attendance at birth contributes to improved maternal outcomes, reinforcing its role as a key strategy for maternal survival in LMICs [9]**.** Despite these benefits, gaps remain in the effective scale-up of BPCR programs in many LMICs, including Nigeria, due to systemic, socio-cultural, and economic barriers [4, 5].

Understanding the perceptions and experiences of midwives, is crucial to improving BPCR uptake. Such insights can inform contextually appropriate interventions and policies aimed at strengthening maternal health services and reducing preventable deaths [13]. This study therefore seeks to explore the barriers and facilitators to BPCR implementation from the perspectives of midwives working in diverse healthcare settings in Ogbomoso, Nigeria.

## Conceptual framework

This study is guided by the Theory of Planned Behavior (TPB), developed by Icek Ajzen (1991), which posits that behavioral intentions are influenced by attitudes, subjective norms, and perceived behavioral control. Midwives' attitudes towards BPCR, their perceptions of institutional expectations (subjective norms), and their confidence in their ability to implement BPCR (perceived control) shape their intention and capacity to promote BPCR practices during antenatal care. The framework is particularly relevant in understanding how socio-cultural, institutional, and personal factors influence midwives' behaviors in the promotion and implementation of BPCR. Applying TPB provides insight into systemic and individual-level interventions necessary to enhance midwives' roles in maternal health.

## Methods

### Research design

The study adopted a qualitative research design, which was ideal for exploring the perceptions, attitudes, and experiences of midwives regarding BPCR. This design allowed for a deeper understanding of how the midwives perceived and navigated BPCR, including any challenges or facilitators they encountered. Semi-structured interviews were utilized to maintain flexibility while allowing for detailed insights into the participants' personal perspectives, practices, and the socio-cultural factors influencing their views on BPCR.

### Research setting

The research was conducted at Bowen University Teaching Hospital, Oyo State General Hospital, and two primary healthcare centers in Ogbomoso: Adebayo Alata Primary Healthcare Centre and Ibrahim Taiwo Primary Healthcare Centre. These facilities played a vital role in providing maternal and child health services. They were chosen for this research due to their significance in offering antenatal care and related maternal health services.

### Study setting

The study was conducted in four healthcare facilities in Ogbomoso, Nigeria:

- Bowen University Teaching Hospital (BUTH) (Tertiary): A private institution known for its diverse clientele and high standards of maternal healthcare.
- Oyo State General Hospital (Secondary): A government-run facility providing comprehensive maternal health services.
- Two Primary Healthcare Centres: Adebayo Alata Primary Healthcare Centre and Ibrahim Taiwo Primary Healthcare Centre, both of which played crucial roles in delivering antenatal and maternal health services at the community level.

### Recruitment setting and study population

#### Study population

The study population consisted of licensed midwives actively engaged in maternal and antenatal care services across selected primary, secondary, and tertiary health facilities in Ogbomoso, Nigeria. These midwives were selected based on their direct involvement in implementing BPCR within their clinical practice.

#### Recruitment process

Participants were recruited using a purposive sampling technique to ensure they had relevant experiences and insights into BPCR. Recruitment was conducted at the antenatal and maternity units of the facilities. Ethical approval and management permissions were obtained before commencing participant recruitment.

#### Sampling size

The sample for this study consisted of a total of 14 midwives selected from each of the four healthcare facilities: Bowen University Teaching Hospital (tertiary), Oyo State General Hospital (secondary), Adebayo Alata Primary Healthcare Centre, and Ibrahim Taiwo Primary Healthcare Centre.

#### Sampling technique

A purposive sampling technique was employed, allowing the researcher to intentionally select participants based on their ability to provide rich, relevant, and specific information about BPCR. This method, a type of non-probability sampling, was commonly used in qualitative research. Its strengths included cost-effectiveness and the ability to target individuals with direct experience related to the study's objectives. However, its limitations included the potential for bias and a lack of generalizability, as participants were not randomly selected. Despite these limitations, purposive sampling was appropriate for this study due to its alignment with the goal of exploring in-depth perceptions and practices related to BPCR.

#### Instrument for data collection

Data for this study were collected through semi-structured indepth interviews, supported by an interview guide, a digital audio recorder, and field notes. This combination provided a balanced approach that ensured flexibility while maintaining a systematic method for capturing detailed insights. The use of field notes also allowed for the observation of non-verbal cues, which enriched the data and helped interpret participants' experiences in greater depth (Polit & Beck, 2016).

The interview guide was divided into two sections:

- Section A: Demographic Information.
  This section gathered relevant background information from participants to contextualize their responses. It collected data on years of experience, educational qualifications, and areas of specialization.

- Section B: Open-ended Questions.
  This section focused on exploring practices, challenges, and facilitators related to BPCR. The questions were tailored to each group to ensure the exploration was relevant to their unique experiences and expertise. These open-ended questions aimed to capture in-depth insights into how midwives perceived BPCR, how it was practiced, and the factors that influenced its effectiveness.

Interviews were conducted in English to ensure comfort during the interview process.

#### Pretesting of the interview guide

The interview guide was pretested with a sample of participants who met the inclusion criteria but were not part of the main study population. This pretest aimed to identify any gaps, ambiguities, or issues with the clarity of the questions to ensure the tool's effectiveness. The pretest involved two interviews, and feedback from these sessions was used to refine the interview guide. Data collected during the pretest were not included in the final analysis.

#### Method of data collection

Semi-structured, audio-recorded interviews lasting between 30 to 60 min were conducted with participants who met the inclusion criteria. These interviews were scheduled during antenatal clinic visits and other relevant settings within the hospitals. Informed consent was obtained from each participant prior to the interview to ensure ethical standards were met. Interviews were conducted in a private setting, chosen by the participant, to maintain confidentiality and foster a comfortable environment that encouraged open and honest responses. The interviews were carried out during work hours but were scheduled in such a way that they did not disrupt patient care or ongoing clinical services. All interviews were conducted by the second author.

The study lasted two months, with interviews continuing until data saturation was reached, ensuring that no new themes or insights emerged. In addition to the audio recordings, field notes were taken to capture non-verbal cues and contextual details, further enriching the data collected.

#### Data analysis and processing

The data collected from the semi-structured interviews were analyzed using a qualitative approach to uncover patterns, themes, and insights related to Birth Preparedness and Complication Readiness (BPCR). The goal of the data analysis was to develop a deeper understanding of the perceptions and experiences of midwives.

Data analysis was conducted using Tesch’s eight-step approach (Tesch, 1991) to ensure a systematic, rigorous process that allowed for the identification of key themes and categories related to the research objectives. The process involved several key stages:

- Getting a sense of the whole:
  All interview transcriptions were read to gain an overall sense of the data. Initial impressions, key ideas, and emerging themes were noted.
  All interviews were conducted by the second author, who also took detailed field notes during and immediately after each session. The interviewer had been trained by the first author, who is experienced in qualitative data collection and analysis. Verbatim transcription of the audio-recorded interviews was likewise carried out by the second author. The completed transcripts were first reviewed by the research team for accuracy and completeness and subsequently sent to an independent analyst for thematic analysis. The analyzed transcripts were then returned to the research team for further review and interpretation.

- Reviewing individual interviews:
  Each interview was reviewed separately. Questions such as, "What is this about?" guided the process.

- Identifying and listing initial themes:
  A list of potential themes was created by grouping recurring ideas, perceptions, and experiences.

- Coding:
  The identified themes were applied to relevant segments of the data. Text segments were coded accordingly.

- Defining themes and categories:
  The themes were refined, clearly defined, and categorized based on their alignment with the research objectives.

- Categorizing and finalizing codes:
  Codes were reviewed, finalized, and organized alphabetically.

- Assembling data:
  Data were systematically organized under each theme and category for detailed examination.

- Recoding:
  Transcriptions were revisited to ensure consistency, and adjustments were made as necessary.

#### Data quality

In qualitative research, data quality was achieved through trustworthiness, ensuring that the findings authentically represented participants’ experiences and perspectives. The criteria of credibility, transferability, dependability, confirmability, and authenticity were met.

##### Credibility

Participants who met the inclusion criteria were selected. Prolonged engagement, member checking, and triangulation were employed.

##### Transferability

Detailed descriptions of context and participants were provided to allow transferability to similar settings.

##### Authenticity

Participants’ words and experiences were presented faithfully and without distortion.

##### Confirmability

In-depth participant experiences were prioritized. Observations were documented, and interviews transcribed immediately to reduce bias. Peer reviews by independent qualitative researchers were also carried out to validate the findings and interpretations, ensuring rigor and reliability in the analysis process. Reflexivity was maintained through a reflexive journal documenting researchers’ assumptions and biases.

### Ethical considerations

The research proposal was submitted to the ethics committees of Bowen University Teaching Hospital (BUTH), General Hospital Sunsun, Adebayo Alata and Ibrahim Taiwo Primary Health Care Centres in Ogbomoso for ethical review and approval. Ethical approval was obtained from Ethics and Research Committee of BUTH (Protocol Number: BUTH/REC-2564). Ethical principles were adhered to throughout the research.

#### Informed consent

Informed consent was obtained from all participants before involvement. Consent forms outlined purpose, benefits, risks, and the use of audio recordings. Participation was voluntary, and participants were informed of their right to withdraw at any time.

#### Anonymity

Anonymity was ensured using pseudonyms and excluding personal identifiers. Only the research team had access to identifying information.

#### Privacy

Privacy was respected by conducting interviews in private settings selected by participants.

#### Publication of results

Participants were informed that results might be published, but anonymity was guaranteed. Findings were presented in aggregate form.

## Findings/results

The analysis yielded two major categories: barriers and facilitators, each comprising several themes grounded in midwives' narratives.

### Participant demographics

The study included 14 licensed midwives drawn from tertiary, secondary, and primary healthcare facilities in Ogbomoso. Participants had between 4 and 28 years of professional experience, with educational qualifications ranging from diplomas in midwifery to bachelor’s degrees in nursing science. Most were actively engaged in antenatal and delivery units at the time of data collection. This demographic information provides context for interpreting their perspectives on BPCR implementation.

### Barriers to implementing BPCR

The accounts of midwives across Ogbomoso painted a sobering picture of the everyday realities that surround childbirth in under-resourced settings. Their words, heavy with empathy and frustration, reveal the immense weight carried by healthcare workers trying to implement BPCR in the midst of poverty, cultural inertia, and systemic failure.

1. Financial constraints and poverty

If there was a single thread woven through every midwife’s story, it was poverty — raw, visible, and persistent. From Bowen University Teaching Hospital to the furthest PHC, midwives recounted how many women arrived at clinics empty-handed, unable to afford delivery kits, medications, or even the ₦500 needed to register for antenatal care.

“Number one is poverty,” (Midwife, Tertiary facility) one midwife said, sighing deeply as if the statement had become her mantra. “Some of them don’t even have ₦100 to buy Dettol, yet we are here talking about emergency transport and blood donors.”

At times, the irony was almost unbearable. A woman could attend every antenatal class, listen attentively, nod at every instruction — but in the end, be undone by an empty wallet and a broken-down motorcycle.

“We tell them to prepare transport for labor,” said another midwife, “and they’ll laugh — ‘Nurse, even to go home from here I’m waiting for okada that will carry me for free!’”

2. Cultural Resistance and Traditional Beliefs

Midwives also had to contend with deep-rooted cultural beliefs that treated childbirth not as a medical event, but as a test of divine will. Among some communities, planning ahead for complications was seen as either unnecessary or, worse, inviting evil.

“They say if you plan for danger, it means you are expecting it,” (Midwife, Secondary facility) said a midwife from General Hospital Sunsun. “One woman told me, ‘Ah ah, Nurse! If I buy blood now, won’t I need it? God forbid!’”

Despite their training, midwives often found themselves tiptoeing around traditions, trying to translate modern obstetric care into languages laced with superstition.

3. Health Illiteracy

Even when there was willingness, understanding was often limited. Health illiteracy remained a huge barrier, particularly in rural PHCs. Many women, midwives said, struggled to grasp the concept of birth preparedness beyond the physical act of showing up at the clinic.

“We show them danger signs and they nod, but some still think you only bleed if someone curses you,” (Midwife,Primary facility) explained a midwife at Adebayo Alata PHC. “It’s not ignorance — it’s just the reality of their world.”

Some midwives resorted to pictures, songs, and even role-playing, hoping that the message would stick better than a long lecture.

4. Inadequate Staffing and Overwhelming Workload

The numbers rarely added up. Two midwives, sometimes one, assigned to care for dozens of pregnant women in a single day — plus emergencies. The result was a constant, grinding overload that made BPCR counseling feel like a luxury.

“We’re just two in the ANC unit. Sometimes I want to sit one woman down and explain everything — but another one is already in labor at the back,” (Midwife, Primary facility) said a visibly fatigued midwife from Ibrahim Taiwo PHC.

BPCR, in such settings, became a list to rush through rather than a conversation to savor.

5. Transportation and Geographical Challenges

Even the best birth plans often fell apart on bad roads. The geography of Ogbomoso’s outskirts — red clay roads, flooding in the rainy season, no ambulances — turned every delivery into a potential crisis.

“She was fully dilated at home, but no one could find a bike,” (Midwife, Primary facility) recounted a midwife. “By the time they reached us, we were praying she didn’t bleed out.”

The unpredictability of transportation continued to undermine the preparedness midwives tried so hard to instill.

6. Limited Male Involvement

A subtle but powerful barrier came from the silence of men — absent partners, passive husbands, or outright resistant ones.

“When a woman says she needs to buy items, the husband may say it’s a waste,” (Midwife, Secondary facility) said a midwife at Bowen. “He doesn’t understand the importance. Sometimes he’s not even around.”

Midwives lamented that without male buy-in, many BPCR efforts faltered before they began.

### Facilitators of BPCR Implementation

Despite the overwhelming odds, there were rays of light stories of creativity, community, and quiet victories that kept midwives going.

1. Visual and Localized Health Education

Where literacy failed, pictures spoke. Midwives found great success using visual tools — posters, drama, songs to explain BPCR concepts. Some even translated materials into local dialects or adapted them into humorous skits.

“I use storytelling a lot,” laughed one midwife. (Midwife, Secondary facility) “Once, I made the women act like they were rushing to hospital without delivery kits. It was so funny, they never forgot it!”

Such methods not only educated but empowered — making BPCR feel relatable, even entertaining.

2. Community and Religious Leaders' Support

Change happened faster when it came from within the community. Some facilities had managed to get local chiefs or church pastors involved in BPCR education.

“We’ve had churches sponsor delivery items,” (Midwife, Primary facility) said a midwife at Ibrahim Taiwo PHC. “When the pastor tells them to plan, they listen more than when we talk!”

This alliance between medical science and traditional authority was proving effective.

3. Family Involvement

Midwives also stressed the importance of involving not just the pregnant woman, but her entire support system.

“I always say, come with your husband or mother-in-law,” shared a midwife. “When the family is informed, planning is easier — and there's less resistance.”

One midwife fondly recalled a man who took notes during every ANC session. “He was more prepared than some of our staff!” she joked. (Midwife, Tertiary facility).

4. Peer Collaboration Among Midwives

Midwives lifted each other up. When the job felt too heavy, they shared tools, techniques, even jokes to get through the day.

“One of us uses drama, another sings. We share what works,” said a Bowen UTH midwife. “Sometimes that’s how we survive — together.”(Midwife, Tertiary facility).

5. NGO Support and Social Services

Where government provisions fell short, NGOs often stepped in with practical support: delivery kits, educational materials, even transport stipends.

“Some NGOs donate packs — baby clothes, pads, gloves. For the poorest women, that’s the only reason they come here,” said a midwife at General Hospital Sunsun. (Midwife, Secondary facility).

This study reveals a complex interplay of barriers and facilitators influencing the implementation of the Birth Preparedness and Complication Readiness (BPCR) strategy in Ogbomoso. Midwives’ narratives highlight pervasive financial constraints and poverty as fundamental obstacles, limiting women’s ability to access essential resources for safe childbirth. Deep-seated cultural beliefs and health illiteracy further complicate efforts, as traditional views often conflict with medical advice and understanding of birth preparedness remains limited. Systemic challenges such as inadequate staffing and overwhelming workloads reduce the quality and depth of BPCR counseling, while geographical and transportation difficulties exacerbate risks during labor. Additionally, limited male involvement undermines family support crucial for effective planning.

Despite these challenges, midwives identify key facilitators that enhance BPCR implementation. Innovative, localized health education using visual aids, storytelling, and drama helps bridge literacy gaps and engage communities. Collaboration with community and religious leaders fosters trust and acceptance, while involving family members strengthens support networks. Peer collaboration among midwives provides emotional and practical resilience, and NGO support supplements scarce government resources with vital materials and services. Together, these themes underscore the multifaceted nature of BPCR implementation and the need for integrated, culturally sensitive, and community-driven approaches to improve maternal health outcomes in resource-limited settings**.**

## Discussion

The challenges faced by midwives in Ogbomoso reveal critical barriers to the effective implementation of BPCR within resource-limited healthcare systems. A major concern identified by midwives is the impact of financial constraints on their ability to deliver comprehensive BPCR education and support. Midwives reported that the economic hardship experienced by many clients limits the success of BPCR initiatives, as pregnant women often cannot afford essential items such as delivery kits, transportation, and antenatal registration fees. This economic barrier undermines the efforts of midwives to encourage early preparation and compliance with BPCR practices. As noted by Ajibade et al. [4], poverty significantly hampers access to maternal healthcare, and this in turn frustrates the efforts of frontline providers like midwives, who are often left to navigate the tension between professional expectations and the socio-economic realities of their clients. These financial challenges limit the effectiveness of health promotion and preparedness counseling and ultimately compromise maternal and neonatal outcomes.

Cultural resistance and traditional beliefs also emerge as formidable obstacles to BPCR uptake. In many communities, childbirth is viewed through a spiritual or fatalistic lens, where planning for complications is perceived as tempting fate or inviting misfortune [7]. This belief system discourages proactive behaviors such as saving money, arranging for blood donors, or planning transport, as some women fear that such preparation might imply an expectation of trouble. Midwives’ accounts mirror findings in similar contexts where superstition and cultural norms create reluctance toward formal health preparations [13].

Health illiteracy further complicates the situation, particularly in rural and underserved populations. Understanding the concepts of danger signs and complication readiness requires a level of health education that is often lacking. According to Gedefa [6], low literacy levels and limited access to health information make it difficult for pregnant women to internalize and act upon BPCR advice. This is compounded using medical jargon and culturally irrelevant health messages that fail to resonate with the community’s lived experiences. Midwives’ use of pictorial aids and local storytelling aligns with best practices identified in the literature for overcoming such communication barriers [13].

In addition to patient-related factors, systemic issues within healthcare facilities contribute significantly to BPCR challenges. Inadequate staffing and overwhelming workloads result in rushed antenatal sessions, leaving little time for individualized BPCR counseling [4]. Overburdened midwives find it difficult to engage women fully or follow up on their preparedness plans, which affects the quality and consistency of care delivery.

Transportation and geographical barriers remain persistent challenges, especially in rural settings characterized by poor infrastructure. Long distances, bad roads, and lack of affordable emergency transport often prevent timely access to health facilities during labor or complications [7]. The absence of reliable ambulances or public transport forces women to rely on informal, often unsafe means to reach care, increasing the likelihood of delays and complications.

The limited involvement of male partners in maternal health decision-making undermines BPCR efforts. Studies show that male partners' support is crucial for ensuring resources and decisions favor maternal health needs [4]. However, many men remain disengaged or lack awareness of BPCR importance, which diminishes the woman’s ability to prepare adequately.

Despite the many barriers faced by midwives in Ogbomoso, there are encouraging facilitators that help promote the successful implementation of BPCR. One of the most effective facilitators highlighted by midwives is the use of visual and localized health education. In contexts where literacy levels may be low, traditional verbal instruction often falls short. Midwives reported significant success in employing visual tools such as posters, dramas, and songs, as well as adapting educational materials into local dialects. Storytelling and role-playing were especially powerful, making the concepts of BPCR more relatable and memorable for pregnant women. This approach resonates with findings from community health education literature, which emphasize that culturally adapted, interactive teaching methods increase understanding and retention [6, 13].

The role of community and religious leaders also emerged as a pivotal facilitator. When trusted local figures, such as chiefs or church pastors, endorse BPCR messages and encourage planning for childbirth, women tend to take the advice more seriously. Midwives recounted instances where churches sponsored delivery items and pastors reinforced the importance of preparation during sermons, thus bridging the gap between modern healthcare practices and traditional belief systems. This community-based endorsement has been shown to improve maternal health outcomes by fostering acceptance and adherence to health interventions [4, 7].

Family involvement is another key facilitator. Midwives emphasized that encouraging women to attend antenatal care sessions accompanied by husbands, mothers-in-law, or other family members leads to better birth preparedness. When the entire support system is informed about potential complications and the importance of planning, resistance decreases, and practical support increases. For example, one midwife fondly recalled a husband who was so engaged that he took notes during every session and helped ensure readiness for delivery. This aligns with broader evidence underscoring the importance of male involvement and family engagement in improving maternal health behaviors and outcomes [4, 13].

Collaboration and peer support among midwives themselves provide essential emotional and practical sustenance in the face of challenging workloads. Sharing teaching techniques, educational tools, and even moments of humor help midwives sustain the quality of BPCR education despite limited staffing and overwhelming demands. This peer collaboration creates an informal but powerful support network that improves morale and service delivery, a factor recognized in health systems research as crucial for maintaining frontline healthcare quality in resource-limited settings [4].

Finally, non-governmental organizations (NGOs) and social services fill critical gaps where government provisions are inadequate. Donations of delivery kits, baby clothes, gloves, and even transportation stipends enable the poorest women to access and benefit from BPCR programs. Midwives acknowledged that, for some women, NGO support is the primary reason they attend clinics and prepare adequately for childbirth. This practical assistance has been documented in many low-resource contexts as a vital enabler of maternal health program success [6, 7].

Together, these facilitators illustrate that while challenges are significant, strategic use of local resources, community engagement, family involvement, peer support, and NGO partnerships can substantially enhance the implementation and impact of BPCR in Ogbomoso.

## Limitation of the study

Although the study included participants with diverse characteristics, findings may however, be specific to Ogbomoso, Oyo State, and may not be applicable to other regions with different healthcare systems, cultural contexts, or levels of resource availability. Nevertheless, the diverse backgrounds of the participants, who were selected from primary, secondary, and tertiary healthcare levels, offer a comprehensive representation of the situation in Oyo State, Nigeria.

## Conclusion

The implementation of Birth Preparedness and Complication Readiness in Ogbomoso is hindered by multifaceted barriers, including pervasive poverty, entrenched cultural beliefs, health illiteracy, inadequate staffing, transportation challenges, and limited male involvement. These obstacles reflect the complex realities of delivering maternal healthcare in resource-constrained settings. However, despite these challenges, facilitators such as culturally adapted health education, the involvement of community and religious leaders, family engagement, peer support among midwives, and NGO contributions provide critical pathways to improving BPCR uptake and effectiveness. Addressing the financial and systemic constraints while leveraging community-based strategies can strengthen maternal health outcomes and reduce preventable childbirth complications. Policymakers, healthcare providers, and community stakeholders must collaborate to sustain and scale these facilitators, ensuring that every pregnant woman receives the support needed for safe childbirth.

## Data Availability

All datasets are available within the manuscript. Any additional information is available on request from the corresponding author.
